# Flat-Cladding Fiber Bragg Grating Sensors for Large Strain Amplitude Fatigue Tests

**DOI:** 10.3390/s100807674

**Published:** 2010-08-16

**Authors:** Aihen Feng, Daolun Chen, Cheng Li, Xijia Gu

**Affiliations:** 1 Department of Mechanical and Industrial Engineering, Ryerson University, Toronto, Ontario, M5B 2K3, Canada; E-Mails: aihan.feng@ryerson.ca (A.F.); dchen@ryerson.ca (D.C.); 2 Department of Electrical and Computer Engineering, Ryerson University, Toronto, Ontario, M5B 2K3, Canada; E-Mail: cheng.li@ryerson.ca (C.L.)

**Keywords:** fiber optic sensor, fiber Bragg grating, fatigue tests

## Abstract

We have successfully developed a flat-cladding fiber Bragg grating sensor for large cyclic strain amplitude tests of up to ±8,000 με. The increased contact area between the flat-cladding fiber and substrate, together with the application of a new bonding process, has significantly increased the bonding strength. In the push-pull fatigue tests of an aluminum alloy, the plastic strain amplitudes measured by three optical fiber sensors differ only by 0.43% at a cyclic strain amplitude of ±7,000 με and 1.9% at a cyclic strain amplitude of ±8,000 με. We also applied the sensor on an extruded magnesium alloy for evaluating the peculiar asymmetric hysteresis loops. The results obtained were in good agreement with those measured from the extensometer, a further validation of the sensor.

## 1. Introduction

In the design of mechanical components, the fatigue properties of materials are of particular importance as in practice 80% to 90% of metal failures arise from fatigue [1]. The fatigue life of components is generally determined from low cyclic fatigue tests at large strain amplitudes and high cyclic fatigue tests at low strain amplitudes. To accurately predict the fatigue life, it is critical to measure the strain in push-pull fatigue tests, especially under the large strain amplitude that induces significant plastic deformation. However, the conventional thin-film strain gauge cannot take large cyclic strain amplitudes; and the extensometer, though capable of taking large strain amplitudes, is relatively large in size, unsuitable for the measurements at boundary of dissimilar materials or for localized strain measurements.

**Figure 1.**
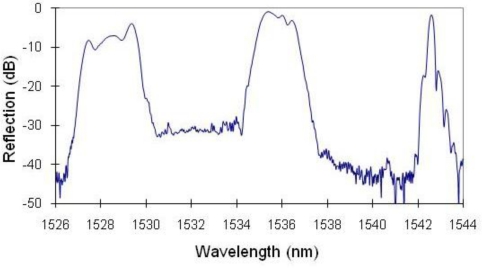
The spectrum shows two severely chirped FBGs and one slightly chirped FBG.

Fiber optic sensors, particularly Fiber Bragg Gratings (FBGs) have become increasingly popular in the last decade due to their wide dynamic range, immunity to electromagnetic interference and their multiplexing capability. FBG sensors, inscribed on an optic fiber of 125 μm in diameter, can be made as short as 2 to 5 mm in length and have been used recently in localized strain measurements, such as on a flip-chip ball grid array that was not accessible for conventional strain gauges [2]. Despite the success of FBGs in strain sensing and structural health monitoring, there are few reports on their applications in fatigue tests of materials, especially in large cyclic strain amplitude tests. When a FBG fiber is bonded to a substrate with epoxy, the substrate strain is transferred to the fiber sensor by the shear stress of the epoxy. Due to the limited contact area between the circular fiber and flat substrate and the shear modulus of the epoxy, a large substrate strain often causes the fiber to slip, rendering the FBG strain reading to be less than the true substrate strain. Using FBGs inscribed on circular fibers, significant chirp in the reflective peaks of the FBG spectra could occur, as shown in Figure 1, when the sample was fatigued for 100 cycles at a strain amplitude of ±5,000 με. This problem has severely limited the applications of FBG sensors in mechanical tests involving large strain amplitudes. The FBG chirp or reflective peak split is mainly due to the uneven slippage between the FBG and the substrate at large strain amplitudes. Reference [3] analyzed in detail the effects of geometric parameters of the adhesive on the substrate strain transfer to FBG, however only at low strain levels. Typically the FBG sensor is used when the strain variation is less than 3,000 με [4]. For example, surface mounted FBG sensors were subject to a mean strain of 1,100 or 2,250 με in the reliability test of the FBG sensors installed on the carbon fiber reinforced polymer cable used in a suspension bridge in Switzerland [5]. In order to overcome this limitation, it is essential to develop a new type of surface-mounted FBG sensors and a new bonding process for the measurement of large dynamic strain amplitudes in fatigue tests and in other material tests such as crack-initiation detection and crack closure evaluation.

In this paper, we report the development of a flat-cladding FBG sensor that significantly increases FBG’s strain measurement range. With a new fiber type and bonding process, we demonstrated that the sensor could measure reliably large strain amplitudes of up to ±8,000 με in aluminum alloys. To the best of our knowledge, this is the highest strain amplitude that surface-mounted FBG sensors can withstand. The calibration and the bonding process of the sensor are described in detail and an application of this sensor in fatigue tests of magnesium alloy samples is also presented.

## 2. Experimental Section

The FBG sensors were fabricated on hydrogen-loaded Flat-cladding fiber (Prime Optical Fiber Corp., Taiwan, ROC) with a collimated KrF excimer laser (Lumonics, model PM 844) emitting laser pulses at 248 nm. The laser beam was focused on a horizontally positioned fiber through a phase mask. The FBGs were apodised with a SINC function with an effective length of 3 mm. All FBGs were annealed at 150 °C for 15 hours after FBG inscription to ensure their long-term stability.

**Figure 2.**
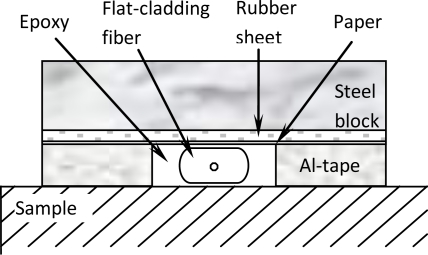
Schematic cross-section diagram of the bonding process.

The flat-cladding fiber has a diameter of 125 μm and a thickness of 83 μm that provides the substrate with a contact width of 93.5 μm, as shown in Figure 2. To ensure that the fiber sensor was bonded to the substrate with a uniform thin layer of epoxy, we used the following procedure: a dog-bone shaped aluminum alloy, used as a base metal, was ground with #320 and then #600 sandpapers in the direction perpendicular to the fiber axis. Two stripes of aluminum-tape of ∼0.10 mm thick were placed alongside the fiber to form a trench of about 1.5 mm wide. After filling the trench with epoxy (part number: 353ND, EPO-TEK), a stripe of non-stick paper and then a thin rubber sheet were used to cover the trench and fiber. A rectangular-shaped weight was placed on the top of the rubber sheet to press the fiber uniformly against the substrate (Figure 2). The assembly was then placed in an oven for curing at 80 °C for 45 minutes.

**Figure 3.**
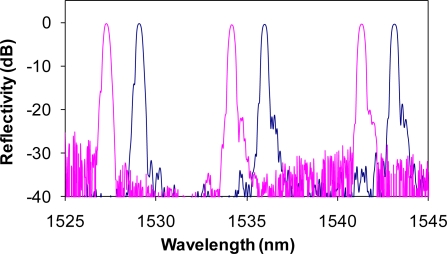
Reflection spectra of a FBG sensor array before (solid line) and after (dashed line) bonded onto the aluminum sample.

The metallic samples tested had a gauge length of 25 mm long (or parallel length of 32 mm) and 6 × 6 mm^2^ in its cross section. The FBG array with three FBG sensors at 1,528.94, 1,536.10 and 1,543.20 nm, respectively, as shown in Figure 3, was bonded on the front flat surface of the sample. The reflective peaks of the FBG sensors showed a high optical signal-to-noise ratio of ∼30 dB. Bonding process caused the blue-shift of the FBG wavelengths by ∼2 nm. However, the bandwidth and the reflectivity of each FBG remained the same.

Strain-controlled, push-pull type fatigue tests were carried out using a computerized fatigue testing system (Instron, Norwood, MA). The sample was tested for 100 cycles at the strain amplitude varying from ±2,000 με to ±9,000 με at an increment of ±1,000 με. An extensometer of 25 mm in gauge length was used to measure the strain and provide feedback to the system under strain control. The wavelength shifts of the FBGs were recorded by a sensor interrogation system (Model si425, Micron Optics) and then were converted to the strain by a gauge factor of 1.25 pm/με. The applied load was recorded simultaneously from an analog output port of the Instron system using an analog-to-digital data acquisition card (Model USB-6215, National Instrument) and was converted into the stress. Thus, both strain and stress values were recorded as a function of time for each fatigue cycle.

## 3. Results and Discussion

### 3.1. Fatigue Test of Aluminum Alloy

In the fatigue tests of below ±4,000 με strain amplitude, the stress dependence on the strain was basically linear, as expected, and the plot of stress *versus* strain curve showed a straight line. As the strain amplitude increased, a hysteresis loop of the stress *versus* strain developed due to the occurrence of cyclic plastic deformation. The hysteresis loops became obvious when the cyclic strain amplitude exceeded ±6,000 με. We measured the width of the hysteresis loop at zero load, *i.e.*, plastic strain amplitude (an important parameter in the low cycle fatigue tests), for each cycle as the cycling progressed.

**Figure 4.**
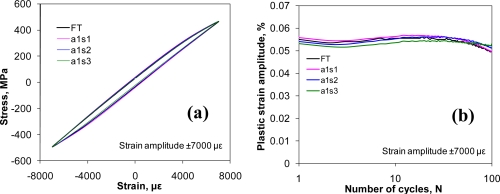
**(a)** FBG sensors measured stress as a function of applied strain at the 10th cycle at ±7,000 με strain amplitudes. **(b)** Plastic strain amplitudes of three sensors at ±7,000 με strain amplitude from the 1st cycle to the 100th cycle. (FT-represents extensometer data, a1s1-refers to Sensor 1 of Array 1 data, so are for a1s2 and a1s3).

Figure 4(a) shows typical hysteresis loops of the 10th cycle measured by three FBG sensors (labeled a1s1, a1s2 and a1s3) in an array on the aluminum sample at a cyclic strain amplitude of ±7,000 με. The hysteresis loop measured from the extensometer was also plotted for comparison (labeled FT). One can see that the results from FBG sensor matched very well with that from the extensometer. When the measured plastic strain amplitudes from the three FBG sensors were compared with that from extensometer for all 100 cycles as shown in Figure 4(b), a good agreement was also observed, not only in the magnitude but also in the trend of plastic strain variation reflecting cyclic hardening-softening behavior of materials. The largest difference in the plastic strain amplitude measured by three FBG sensors was about 0.003%, which was only 0.43% of the cyclic strain amplitude of ±7,000 με. This clearly validated that flat-cladding FBG sensors can be used to measure large strain amplitudes in fatigue tests.

**Figure 5.**
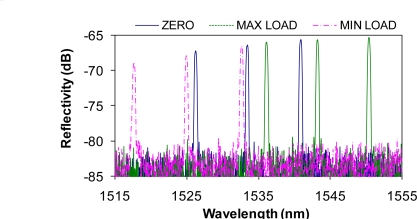
The spectra of the array at three different loads after 100 cycles at ±7,000 με strain amplitude.

In order to determine if the bonding between fiber sensor and substrate was still intact, we measured the spectra of the FBG array at zero, maximum, and minimum load after each cyclic loading test. The reflective spectra of the array after 100 cyclic loads at ±7,000 με strain amplitude are shown in Figure 5. We see no change in the relative wavelength spacing between the sensors at three loads, indicating no slippage between the flat-surface sample and optical fiber. In contrast to Figure 1, we also see no bandwidth broadening in any of the three sensors, indicating that no chirp occurred in any of the FBGs, *i.e.*, the strain has been transferred uniformly along the fiber. The compression of the sample at the minimum load did reduce the reflectivity of the FBG sensors by 1 to 2 dB as compared with no load or in the tensile condition. Nevertheless, the reduced reflectivity would not affect our strain measurement since the strain was determined by the wavelength shift, not by the intensity of the FBG peak.

We also tested the same sample at cyclic strain amplitudes of ±8,000 με for 100 cycles. The measured plastic strain amplitudes from the three FBGs agreed with that from the extensometer as well. However, the difference in the plastic strain amplitudes from three FBG sensors increased to 0.019%, *i.e.*, 1.9% of the strain amplitude at ±8,000 με. We also observed bandwidth broadening of the FBG peaks by 10 to 15% when compared with the bandwidth of the FBGs after 100 cycles under ±7,000 με. This bandwidth broadening indicated that strain transfers started to become non-uniform along the fiber as the strain amplitude increased to ±8,000 με. The aluminum sample broke at the 9th cycle in the ±9,000 με strain amplitude tests. Based on these data, we can conclude that the upper limit of the strain measurements using the flat-cladding FBG sensor on this type of sample was around ±8,000 με. This limit could be further extended by the improvement of bonding material and process, such as using an epoxy with high shear modulus.

### 3.2. Fatigue Test of Magnesium Alloy

To further validate the flat-cladding FBG sensor, we also bonded a sensor array on an extruded AZ31 magnesium alloy sample and conducted fatigue tests. The magnesium alloy is a lightweight structural material with a peculiar and large asymmetric hysteresis loop under cyclic loading [6]. In the strain-controlled fatigue test, we observed plastic deformation even at the low cyclic strain amplitude of ±2,000 με and the deformation became more prominent with increasing strain amplitude.

**Figure 6.**
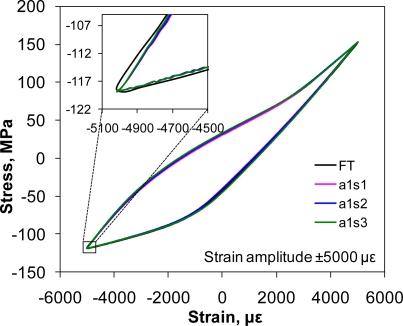
Hysteresis loops of the AZ31 magnesium alloy at the 10th cycle at a strain amplitude of ±5,000 με. The insert shows the enlarged compression-decompression corner.

Figure 6 compares the hysteresis loops measured from three FBG sensors on an array bonded on an extruded AZ31 magnesium alloy sample with the hysteresis loop measured from the extensometer at ±5,000 με strain amplitude. One can see that the stress-strain curves measured by all the three FBG sensors matched almost perfectly with that from the extensometer. The large asymmetric loop, especially the plastic deformation in its compressive and decompressive/tensile phases helps to understand the unusual microscopic change (*i.e.*, twinning-detwinning) of magnesium alloy during fatigue deformation.

## 4. Conclusions

We have successfully developed a new FBG-based sensor for the measurements of large cyclic strain variations. The large contact area between the flat-cladding and flat substrate, along with the application of a new bonding process, has significantly increased the bonding strength, which in turn provided a proper transfer of the strain from the substrate to the optical fiber. Our test data showed that the flat-cladding FBG sensors can be used to measure cyclic strain amplitudes as large as ±8,000 με in dynamic fatigue tests under the current test conditions. This represents a remarkable increase of the strain measurement that could make the FBG sensor a vital tool in the low cycle fatigue tests at large strain amplitudes. To the best of our knowledge, other than the FBG sensors embedded inside carbon fiber reinforced polymer, we are not aware of any surface-mounted FBG sensors that can withstand such high cyclic strain amplitudes. Furthermore, due to its small size, the FBG sensor could be used to measure the localized strain variations, for example, in the welding joints, detect fatigue crack initiation, and determine crack closure effect during fatigue crack propagation. We reported recently an application of this sensor to measure the localized strain variation in a friction-stir-welded sample [7]. Nevertheless, the development of the sensor including high strain amplitude tests and bonding process was not reported. We believe that this sensor would provide a new tool for the researchers and engineers in the development and tests of new materials and mechanical structure where the tests and monitoring of the large cyclic strain amplitudes are critically needed.
